# Laminar Pattern of Projections Indicates the Hierarchical Organization of the Anterior Cingulate-Temporal Lobe Emotion System

**DOI:** 10.3389/fnana.2019.00074

**Published:** 2019-07-31

**Authors:** Honami Sakata, Yuri Kim, Masafumi Nejime, Naho Konoike, Shigehiro Miyachi, Katsuki Nakamura

**Affiliations:** Cognitive Neuroscience Section, Primate Research Institute, Kyoto University, Inuyama, Japan

**Keywords:** ACC, primate, retrograde tracing, hierarchical organization, temporal pole

## Abstract

The anterior cingulate cortex (ACC), surrounding the genu of the corpus callosum, plays important roles in emotional processing and is functionally divided into the dorsal, perigenual, and subgenual subregions (dACC, pgACC, and sgACC, respectively). Previous studies have suggested that the pgACC and sgACC have distinctive roles in the regulation of emotion. In order to elicit appropriate emotional responses, these ACC regions require sensory information from the environment. Anatomically, the ACC has rich connections with the temporal lobe, where the higher-order processing of sensory information takes place. To clarify the organization of sensory inputs into the ACC subregions, we injected neuronal tracers into the pgACC, sgACC, and dACC and compared the afferent connections. Previously, we analyzed the afferent projections from the amygdala and found a distinct pattern for the sgACC. In the present study, the patterns of the afferent projections were analyzed in the temporal cortex, especially the temporal pole (TP) and medial temporal areas. After tracers were injected into the sgACC, we observed labeled neurons in the TP and the subiculum of the hippocampal formation. The majority of the labeled cell bodies were found in the superficial layers of the TP (“feedforward” type projections). The pgACC received afferent projections from the TP, the entorhinal cortex (EC), and the parahippocampal cortex (PHC), but not from the hippocampus. In each area, the labeled cells were mainly found in the deep layers (“feedback” type projection). The pattern for the dACC was similar to that for the pgACC. Previous studies suggested that the pgACC, but not the sgACC receive projections from the dorsolateral prefrontal cortex (DLPFC). These data suggest that the sgACC plays crucial roles for emotional responses based on sensory and mnemonic inputs from the anterior temporal lobe, whereas the pgACC is more related to the cognitive control of emotion.

## Introduction

The anterior cingulate cortex (ACC) has been implicated in emotional processing and is divided into three subdivisions: the subgenual (sgACC), pregenual (pgACC), and dorsal (dACC) divisions (Drevets and Raichle, 1998; Bush et al., 2000; Ochsner and Gross, 2005; Etkin et al., 2011; Caruana et al., 2018). Recent studies have demonstrated that the pgACC and sgACC have distinct roles in the regulation of emotion. Hyperactivity of the sgACC has been observed in patients with depression (Drevets, 2000; Myers-Schulz and Koenigs, 2012), whereas pgACC activity is associated with positive affect (Myers-Schulz and Koenigs, 2012) and effectiveness of antidepressants (Mayberg et al., 1997; Godlewska et al., 2018). In non-human primates, inactivation of the sgACC decreased experimentally-induced negative emotions, and inactivation of the pgACC showed the opposite effects (Wallis et al., 2017). Furthermore, overactivation of the sgACC, but not the pgACC, blunted reward-elicited anticipatory and motivational arousal (Alexander et al., 2019b). These findings suggest that the pgACC and sgACC may regulate emotion in a complementary manner. In rodents, the prelimbic (PL) and infralimbic (IL) regions of the medial prefrontal cortex respectively correspond to the pgACC and sgACC in humans. However, previous studies have suggested that effects of PL and IL activations in rodents have opposite effects to the activation of the pgACC and sgACC in humans and non-human primates (Vidal-Gonzalez et al., 2006; Baldi and Bucherelli, 2015; Alexander et al., 2019a). Roles of the rodent PL/IL may be different from those of the pgACC and sgACC in primates.

In order for these ACC regions to regulate emotional response adequately, sensory information about the environment is indispensable. Visual information is especially crucial for humans and other primate species for both expression of appropriate emotional responses and comprehension of others’ facial expressions. Recent studies have shown that the ACC responds to emotional facial stimuli (Gotlib et al., 2005; Ito et al., 2017). Recently, we have recorded neuronal activities in the dACC and pgACC of monkeys, and found responses to emotional visual stimuli in both areas (Konoike and Nakamura, 2013). To date, neuroanatomical studies have shown that the ACC receives strong cortical and subcortical inputs from the anterior temporal lobe (Moran et al., 1987; Vogt and Pandya, 1987; Amaral et al., 1992; Carmichael and Price, 1995; Barbas et al., 1999; Ghashghaei and Barbas, 2002; Saleem et al., 2008; Morecraft et al., 2012; Joyce and Barbas, 2018). Previously, we analyzed the organization of afferent inputs from the amygdala (Kim et al., 2018) to the pgACC, sgACC, and dACC and found that the sgACC received rich inputs from the accessory basal and lateral nuclei, in which neuronal responses selective for face and those selective for negative stimuli were recorded (Leonard et al., 1985; Nishijo et al., 1988; Boll et al., 2011). Besides the amygdala, the temporal cortex, especially its anterior and medial regions, is also crucial for the recognition of faces and other complex visual stimuli (Perrett et al., 1982; Kobatake and Tanaka, 1994; Nakamura et al., 1994; Browning et al., 2010). Damage to the anterior temporal cortex causes prosopagnosia (Nakamura and Kubota, 1996). The temporal pole (TP) is also related to social and emotional functions (Olson et al., 2007; Pehrs et al., 2017).

In the present study, we used the same animals that were used in our previous work (Kim et al., 2018) to compare the neuronal inputs to the three ACC regions from the anterior temporal cortex, including the hippocampus. In addition to the distribution pattern of the labeled neurons, the laminar distribution in each area was also examined to determine the hierarchical organization of these areas and the direction of the flow of information.

## Materials and Methods

### Animals

Seven Japanese macaques (*Macaca fuscata*) of both sexes were used in this study. The experiments were conducted according to the Guide for the Care and Use of Laboratory Primates of the Primate Research Institute, Kyoto University (2010). This study was approved by the Animal Welfare and Animal Care Committee of the Primate Research Institute, Kyoto University.

### Tracer Injections

Detailed descriptions of the procedures used in this study are provided elsewhere (Kim et al., 2018). Briefly, combinations of 2–4 different retrograde tracers (0.2–0.6 μL each) were pressure injected through a 5-μL microsyringe. The following tracers were used: fluoro-ruby (10,000 molecular weight (MW); Thermo Fisher Scientific Inc., Waltham, MA, USA), fluoro-emerald (10,000 MW; Thermo Fisher Scientific Inc., Waltham, MA, USA), lucifer yellow dextran (10,000 MW; Thermo Fisher Scientific Inc., Waltham, MA, USA), and cholera toxin B subunit (Sigma-Aldrich Corporation, St. Louis, MO, USA). Injection sites were chosen based on magnetic resonance images taken prior to the surgery (Table 1).

**Table 1:** Number of labeled neurons in the temporal areas.

Case	Tracer	Leak	Number of labeled neurons (%)							
			TPC	EC	PRC	PHC	H	IT	ST	Total
sgACC
N549l-sg	CTB (1%, 0.6 μl)	no	724 (77%)	1 (0%)	0 (0%)	0 (0%)	173 (18%)	0 (0%)	42 (4%)	940 (100%)
M2303r-sg	FE (10%, 0.3 μl)	no	31 (15%)	5 (2%)	4 (2%)	11 (5%)	146 (70%)	1 (0%)	11 (5%)	209 (100%)
N549r-sg	LYD (2%, 0.4 μl)	str	132 (64%)	15 (7%)	4 (2%)	3 (1%)	45 (22%)	1 (0%)	5 (2%)	205 (100%)
M2307r-sg	FE (10%, 0.3 μl)	str	428 (43%)	60 (7%)	30 (3%)	67 (7%)	328 (33%)	3 (0%)	70 (7%)	986 (100%)
M2452r-sg	FR (2%, 0.2 μl)	str	152 (22%)	47 (7%)	21 (3%)	50 (7%)	386 (57%)	1 (0%)	22 (3%)	679 (100%)
pgACC
M2307r-pg	FR (10%, 0.3 μl)	no	81 (34%)	41 (17%)	2 (1%)	27 (11%)	2 (1%)	7 (3%)	75 (32%)	235 (100%)
M2303r-pgd	LYD (10%, 0.3 μl)	no	353 (35%)	168 (17%)	70 (7%)	296 (30%)	17 (2%)	16 (2%)	82 (8%)	1,002 (100%)
M2303r-pgv	FR (10%, 0.3 μl)	str	224 (18%)	182 (15%)	158 (13%)	265 (21%)	348 (28%)	27 (2%)	51 (4%)	1,255 (100%)
M2305r-pg	FR (10%, 0.3 μl)	str	419 (18%)	752 (33%)	656 (28%)	270 (12%)	157 (7%)	5 (0%)	48 (2%)	2,307 (100%)
dACC										
N497r-d	FE (10%, 0.3 μl)	no	60 (65%)	9 (10%)	7 (8%)	1 (1%)	0 (0%)	0 (0%)	16 (17%)	93 (100%)
N509l-d	CTB (1%, 0.5 μl)	no	55 (15%)	99 (28%)	29 (8%)	14 (4%)	2 (1%)	2 (1%)	159 (44%)	360 (100%)
N549r-d	FR (2%, 0.4 μ)	no	55 (58%)	4 (4%)	4 (4%)	9 (10%)	0 (0%)	0 (0%)	22 (23%)	94 (100%)
N549l-d	FE (10%, 0.4 μl)	area 8B	223 (36%)	174 (28%)	71 (11%)	120 (19%)	1 (0%)	3 (0%)	31 (5%)	623 (100%)
M2452r-d	FE (10%, 0.2 μl)	str	90 (7%)	216 (18%)	720 (59%)	158 (13%)	36 (3%)	1 (0%)	5 (0%)	1,226 (100%)

*IT, inferior temporal cortex; ST, superior temporal cortex including the dorsal bank of the superior temporal sulcus and the superior temporal gyrus*.

### Histological Procedures

At 3–4 weeks after tracer injections, the animals were deeply anesthetized with sodium pentobarbital (40 mg/kg) and transcardially perfused with phosphate buffered saline (PBS) and 10% formalin. Brains were immersed in PB containing 30% sucrose, and 50-μm thick coronal sections were cut serially. Every 8th section was immuno-histochemically stained for each tracer by using the standard avidin-biotin-peroxidase complex method. The sections were mounted on glass slides, air-dried, counterstained with neutral red, and covered with a coverslip.

The cortical areas were defined based on the cytoarchitecture (Amaral et al., 1987; Vogt et al., 1987; Carmichael and Price, 1994, 1995; Kondo et al., 2003; Suzuki and Amaral, 2003; Saleem et al., 2007; Morecraft et al., 2012; Kim et al., 2018). For parcellation of the perirhinal and parahippocampal areas, we mainly followed the definitions of Suzuki and Amaral (2003). TP areas were defined according to Carmichael and Price (1995; see also Moran et al., 1987).

### Data Analysis

The number of labeled neurons in each area was counted in every 16th section (800 μm apart). The areas subjected to the present analysis were the TP agranular region (TPag), dorsal dysgranular region (TPdgd), ventral dysgranular region (TPdgv), granular region (TPg), entorhinal cortex (EC), perirhinal cortex (PRC: areas 35 and 36), parahippocampal cortex (PHC: TH and TF), and hippocampus (H: subiculum, CA1, CA2, CA3, and dentate gyrus). In addition, the number of labeled neurons was counted in the inferior temporal cortex (IT), including both the inferior convexity and the inferior bank of the superior temporal sulcus, and the superior temporal cortex (ST), including the dorsal bank of the superior temporal sulcus and the superior temporal gyrus. For all areas (except H), the percentage of the neurons in the superficial (supragranular) layers, “superficial ratio,” was calculated if there were 20 or more labeled neurons in the area. For more detailed analysis of the TP, the superficial ratio was calculated for TPa, TPdgd, TPdgv and TPag, in every 8th section.

## Results

### Injection Sites and TP Architecture

**Figure 1:**
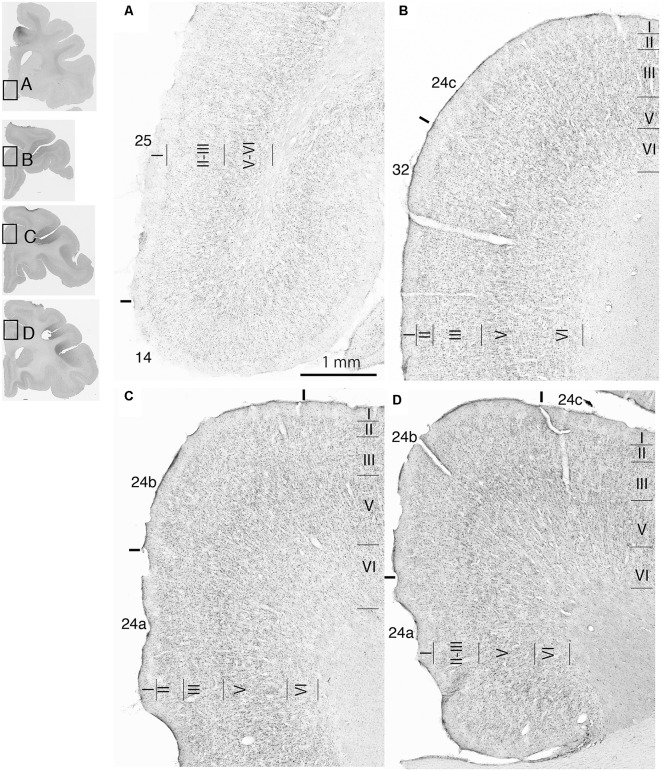
Photomicrographs of the anterior cingulate cortex (ACC) regions. Subgenual ACC (sgACC) corresponds to area 25 **(A)**. Pregenual ACC (pgACC) includes area 32 **(B)** and the pregenual portion of area 24 **(C)**. Dorsal ACC (dACC) is the supragenual portion of area 24 above sgACC **(D)**.

**Figure 2:**
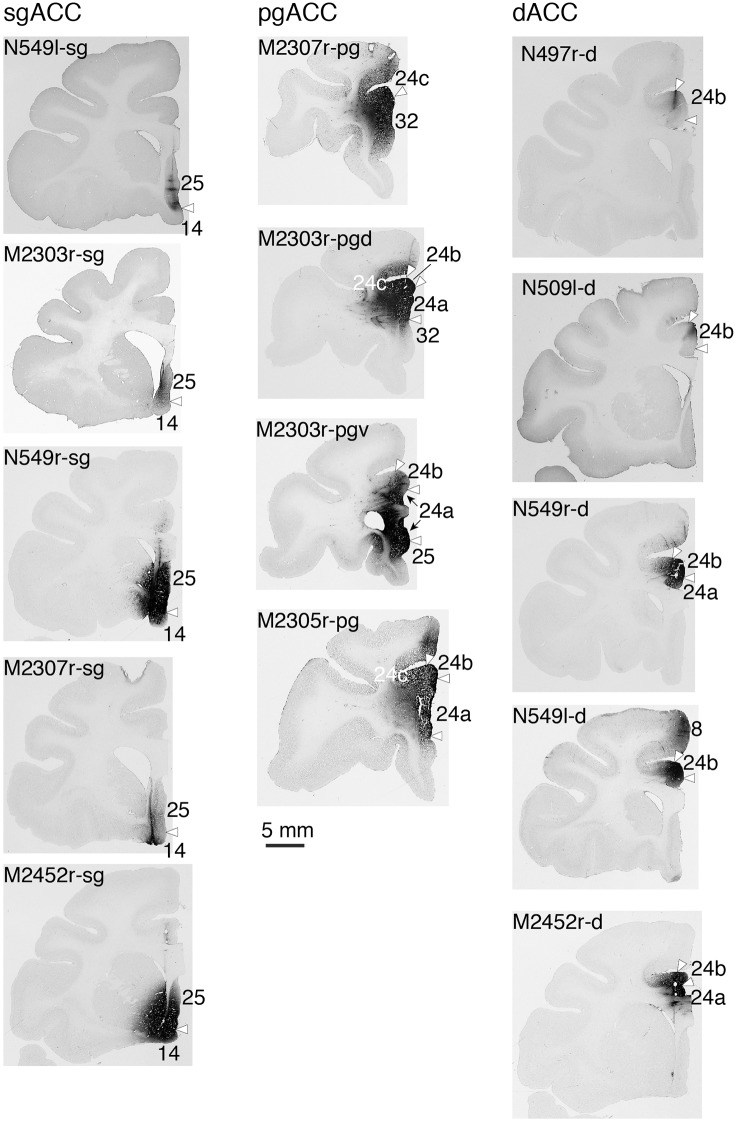
Photographs of the brain sections through the center of injection.

**Figure 3:**
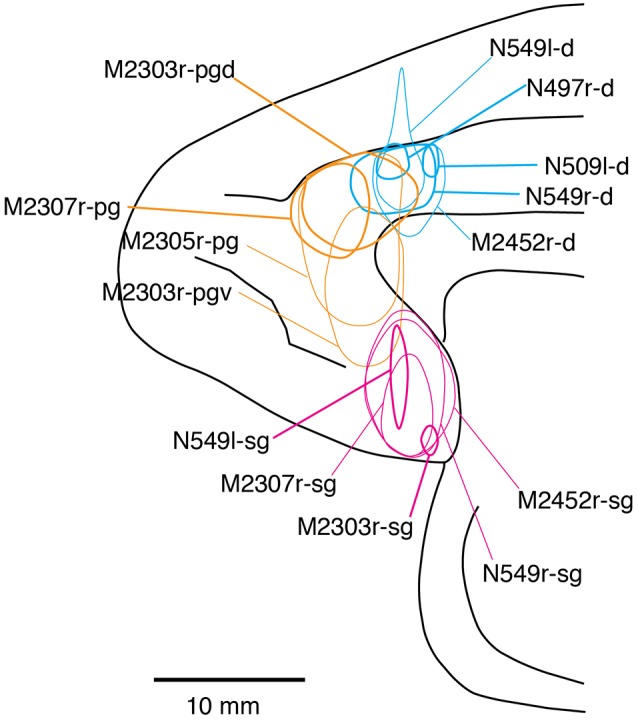
Tracer injection sites shown from the medial view of the brain. Actual injections were in either the left or right hemispheres. The sgACC, pgACC, and dACC injection sites are delineated in magenta, orange, and cyan, respectively. Thick lines indicate the injections that were confined to the targeted area, whereas the thin lines indicate the injections that spread to the neighboring structures (i.e., the striatum).

Fourteen tracer injections were made in eight hemispheres that targeted the sgACC, pgACC, or dACC. The three ACC regions were defined based on location and cytoarchitecture. The sgACC is the medial wall cortex below the genu of the callosal body, corresponding to area 25. In Nissl stained sections, this area has essentially three layers: (1) layer I: (2) a lightly stained superficial layer (layers II–III); and (3) a deeply stained deep layer (layers V–VI; Figure 1A). The pgACC is the cortex anterior to the genu of the callosal body, including both area 32 and the pregenual portion of area 24. Area 32 is an agranular cortex in which layer V has characteristic horizontal striations (Figure 1B). Area 24b can be distinguished by the vertical arrangement of layer V neurons. Caudal to area 32 is area 24a, in which layers II and III are difficult to be distinguished, and no vertical or horizontal striations of the cells can be seen in layer V (Figure 1C). The dACC is the cingulate gyrus above the callosal body at the same anteroposterior level as the sgACC. It includes areas 24a and 24b, but actual injections sometimes spread into the adjacent area, area 24c, which has no clear vertical striations like those in 24b, but has aggregates of medium sized neurons in layer V (Figure 1D). Photographs of the sections through the injection sites are shown in Figure 2, and the approximate locations of the injection sites are summarized in Figure 3. The actual injection site in each case was in either the right or left hemisphere. In seven cases (thin lines in Figure 3), the injection spread to the mediodorsal edge of the caudate head (M2303r-pgv, M2305r-pg, M2452r-d), to the medial most part of the ventral striatum (N549r-sg, M2307r-sg, M2452r-sg), or leaked into the cortex above the cingulate sulcus (area 8B, N549l-d). In other cases (thick lines in Figure 3), the injection was mostly confined to the target area. In N549l-sg and M2303r-sg (sgACC cases), injections were largely confined to area 25 and slightly spread into area 14. In M2303r-sg, the tracer was injected into the deep layer and moderately spread to the superficial layers (Figure 3). Tracers that were injected into the M2303r-pgd (pgACC) spread caudally and encroached the dACC; however, the pattern of the retrograde labeling was similar to that of other pgACC cases. Among the dACC injections, the injection site in N509l-d was clearly more caudal to those of N497r-d and N549r-d.

**Figure 4:**
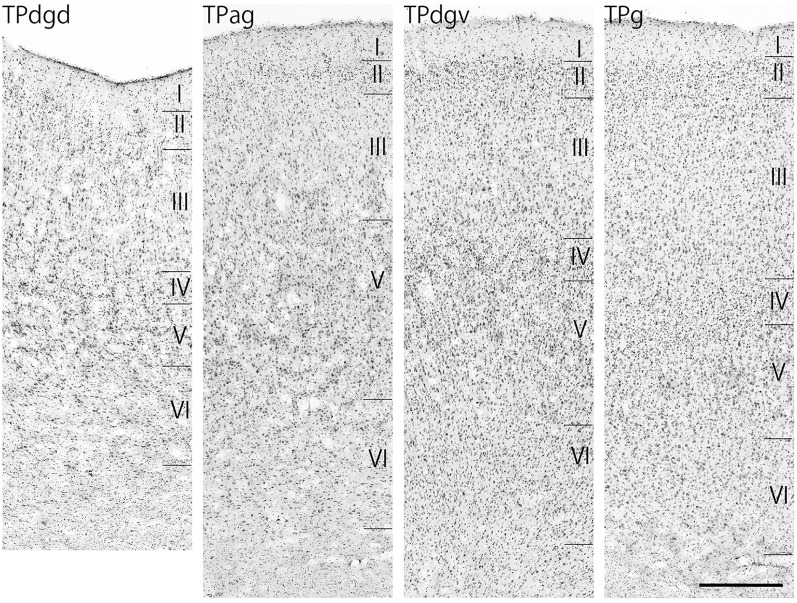
Nissl-stained sections of the four temporal pole (TP) regions. Scale bar: 500 μm. TPdgd, dorsal dysgranular region of the TP; TPag, agranular region of the TP; TPdgv, ventral dysgranular region of the TP; TPg, granular region of the TP.

The TP was divided into four subdivisions: a granular region (TPg), dorsal and ventral dysgranular regions (TPdgd and TPdgv), and an agranular region (TPag; Figure 4).

### Projections to the sgACC

**Figure 5:**
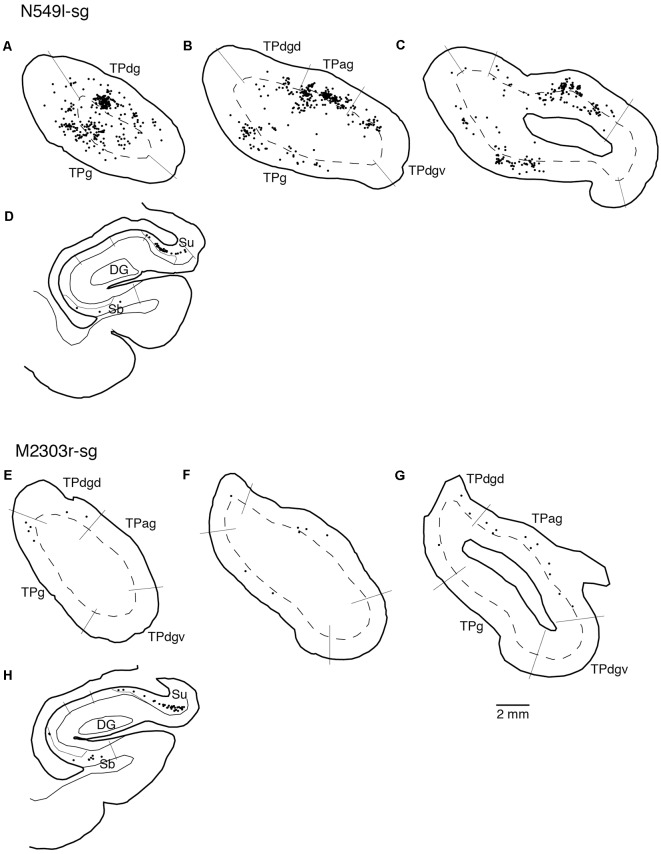
Distribution of the labeled neurons in the TP and hippocampal formation after injections into the sgACC in two cases (N549l-sg and M2303r-sg). The sections through the TP are arranged from anterior to posterior **(A–C** and **E–G)**. **(D,H)** Sections through the hippocampus. Broken lines indicate the internal granular layer or border between layer III and layer V. DG, dentate gyrus; S (b, u), subiculum (body and uncal portion); TP (ag, dgd, dgv, g), temporal pole cortex (agranular, dorsal dysgranular, ventral dysgranular, and guranular subdivision).

**Figure 6:**
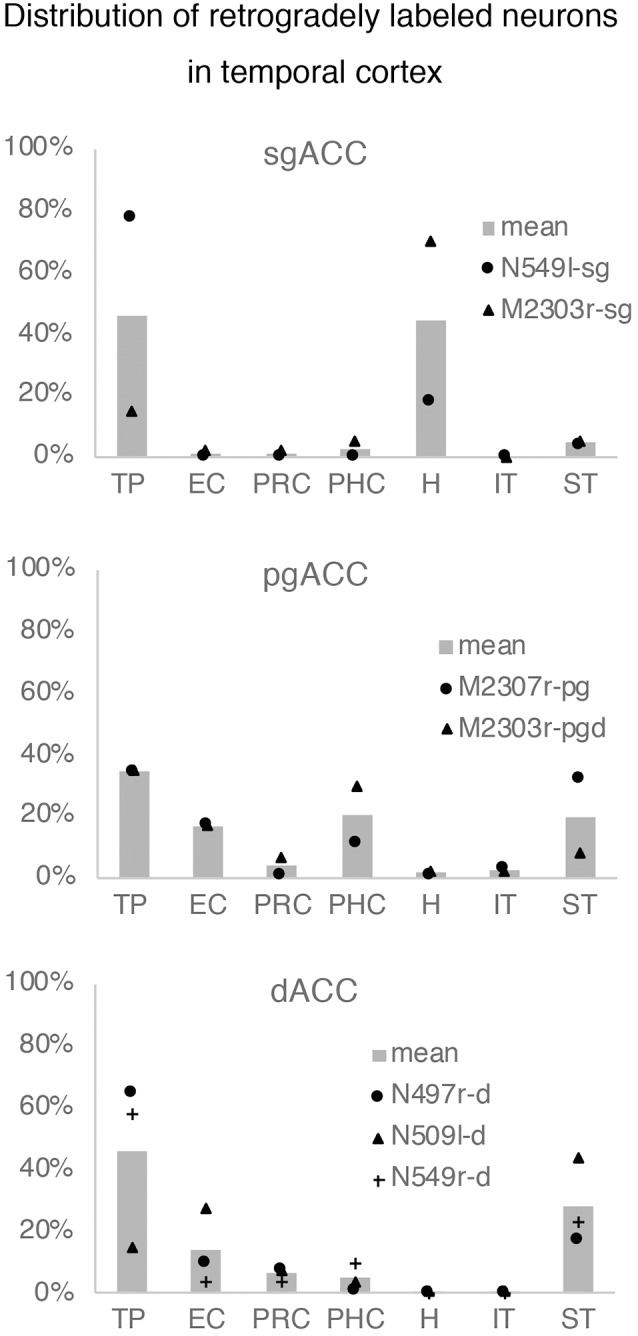
The number of retrogradely labeled neurons in each area within the temporal cortex. The graphs show the results of the cases in which thetracer injection was well confined to the targeted area (see “Results” section).EC, entorhinal cortex; H, hippocampus; IT, inferior temporal cortex; PHC,parahippocampal cortex; PRC, perirhinal cortex; ST, superior temporal cortex; TP, temporal pole.

**Figure 7:**
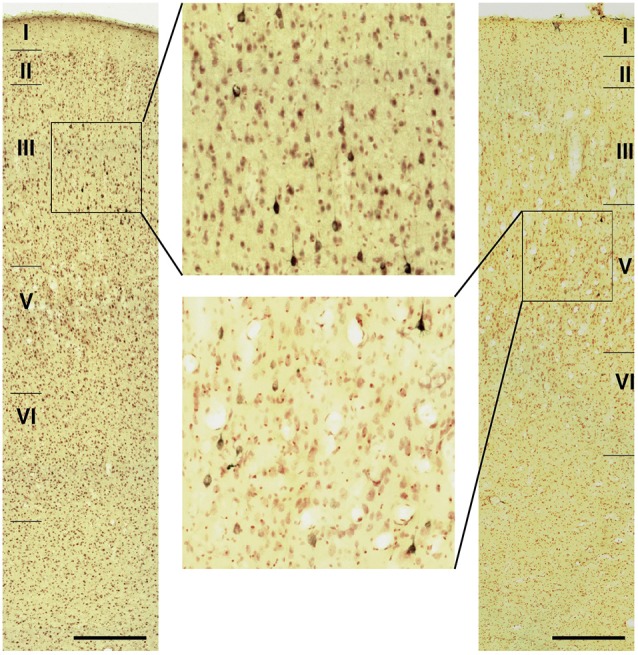
Photomicrographs of the TP (TPag) in cases N549l-sg (left) andM2303r-pgd (right). The areas including labeled neurons are enlarged. Scale bar: 500 μm.

Neuronal tracers were injected into the sgACC in five cases (Figures 2, 3). In two cases (N549l-sg and M2303r-sg), the injection was well confined to the sgACC. In these cases, a large majority of the labeled neurons were found in the TP and the subiculum (Table 1, Figures 5, 6). In case N549l-sg, 77% and 15% of the labeled neurons were found in the TP and subiculum, respectively. Among the TP areas, the TPag accommodated about half of the labeled cells. Another region of dense labeling was observed in the TPg. In M2303r-sg, the distribution pattern was essentially the same as in N549l-sg; however, more neurons were labeled in the subiculum than in the TP. A small number of labeled neurons were scattered in the EC, PRC, and PHC (Table 1). Essentially no neuronal labeling was observed in the IT (including area TE) in either of these animals, and a small number of labeled neurons was scattered in the ST. In the TP of both animals, more than two thirds of the labeled neurons were found in the superficial layers (layers II and III; Figure 7 left, Table 2). For more detailed analysis of the laminar distribution in the TP, the superficial ratio was calculated in each subdivision of the TP (Table 3). The ratio was over 80% in the TPg, whereas it was around 50% in other subdivisions. In the three other cases, the tracers diffused laterally into the medial portion of the ventral striatum. Nevertheless, the distribution pattern of neuronal labeling in the medial temporal/temporal pole region was similar to those of the first two cases: the majority of the labeled neurons were found in the TP and in the subiculum. In N549r-sg and M2307r-sg, more than half (56% and 62%, respectively) of the labeled TP neurons were located in the superficial layers. Conversely, in M2452r-sg, only 32% were located in the superficial layers.

**Table 2:** Ratios of labeled neurons in the superficial layers.

Case	Leak	Superficial ratio					
		TPC	EC	PRC	PHC	IT	ST
SgACC							
N549l-sg	no	67%					90%
M2303r-sg	no	68%					
N549r-sg	str	56%					
M2307r-sg	str	62%	0%	40%	45%	67%	81%
M2452r-sg	str	32%	2%	38%	4%		27%
pgACC							
M2307r-pg	no	22%	0%		4%		75%
M2303r-pgd	no	18%	1%	20%	16%		55%
M2303r-pgv	str	21%	5%	56%	26%	96%	88%
M2305r-pg	str	31%	6%	79%	5%		50%
dACC							
N497r-d	no	5%					75%
N509l-d	no	9%	0%	21%			76%
N549r-d	no	13%					59%
N549l-d	area 8B	6%	1%	3%	0%		13%
M2452r-d	str	12%	17%	81%	22%		

*The superficial ratio was calculated if there were 20 or more neurons in each area*.

**Table 3:** Superficial ratios in subregions of the TP.

Case	Superficial ratio			
	TPag	TPdgd	TPdgv	TPg
sgACC				
N549l-sg	58%	50%	47%	81%
M2303r-sg	54%	(86%)	-	(91%)
pgACC				
M2307r-pg	9%	-	(0%)	32%
M2303r-pgd	7%	34%	(19%)	38%
dACC				
N497r-d	0%	(15%)	(0%)	17%
N509l-d	2%	-	-	-
N549r-d	6%	(12%)	-	-

*Parenthesis indicates the total number of labeled neurons in the area was 10–19. “-” indicates the total number was less than 10. The ratio was calculated only for the cases with well-confined injection in the target region*.

### Projections to the pgACC

**Figure 8:**
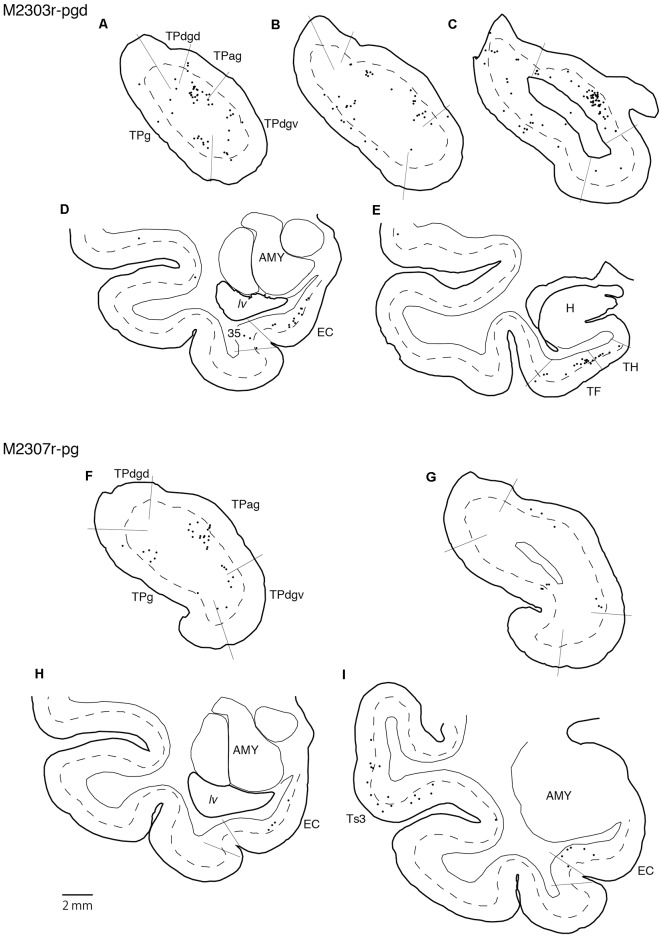
Distribution of the labeled neurons in the TP and medial temporal cortex after injections into the pgACC (cases M2303r-pgd and M2307r-pg). **(A–C,F,G)** Sections through the TP. **(D,E,H,I)** Sections through the medial temporal cortex. 35, area 35 of perirhinal cortex; AMY, amygdala; EC, entorhinal cortex; H, hippocampus; lv, lateral ventricle; TE, area TE; TF, area TF; TH, area TH. Other conventions are as shown in Figure 4.

Tracers were injected into the pgACC in four cases (Figures 2, 3). In two cases (M2307r-pg and M2303r-pgd), the injections were largely confined to the targeted cortex, but we also observed slight spreading of the tracers into the white matter. In these cases (M2307r-pg and M2303r-pgd), many labeled neurons (34% and 35%, respectively) were found in the TP (Table 1, Figures 6, 8). Additionally, in M2307r-pg, 32% of labeled neurons were found in the ST (area Ts3). In both monkeys, an appreciable number of neurons were labeled in the EC and PHC, but very few neurons were labeled in the subiculum or other regions in the H. In contrast to sgACC injections, labeled TP neurons were mainly observed in the deep layers following pgACC injections (layers V and VI; Figure 7 right, Table 2). The superficial ratio was low in all the TP subdivisions (Table 3). In the EC and PHC, like in the TP, a large majority of the labeled neurons was in the deep layers. In the other cases (M2303r-pgv and M2305r-pg), the tracers spread medially into the medial caudate nucleus and a small number of striatal neurons was labeled. Caudoventrally, the tracer also spread to the cortex, ventral to the genu of the callosal body, especially in M2303r-pgv. In these cases, more neurons were labeled in the superficial layers of the PRC than those in M2307r-pg and M2303r-pgd (Tables s 1, 2). Furthermore, in M2303r-pgv, many labeled neurons were found in the H. The ST contained only a small number of labeled neurons in both cases.

### Projections to the dACC

**Figure 9:**
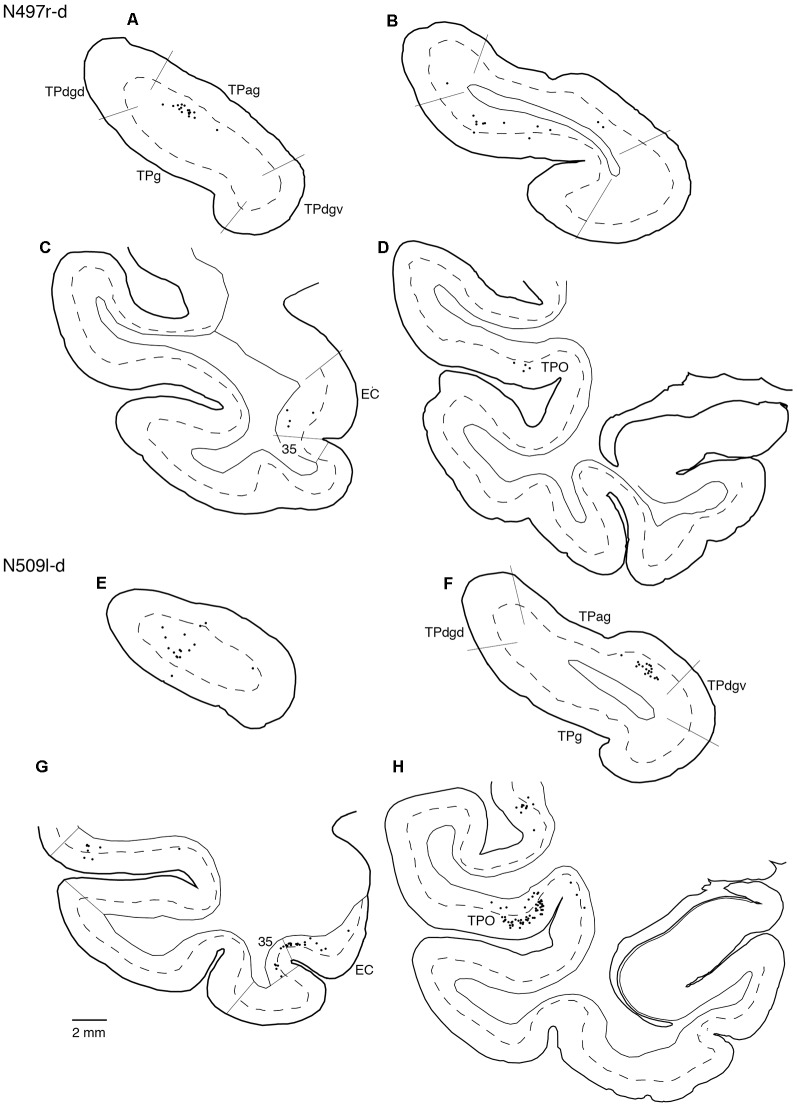
Distribution of the labeled neurons in the TP **(A,B,E,F)** and other temporal cortical areas **(C,D,G,H)** after injections into the dACC (cases N497r-d and N509l-d). The conventions are the same as shown in Figures 4, 5.

Tracers were injected into the dACC in five cases (Figures 2, 3). In three of these cases (N497r-d, N509l-d, and N549r-d), the injections were well confined to the cingulate cortex. Among these three cases, the injection site in N509l-d was apparently more caudal compared to those in the other two cases (Figures 2, 3). N497r-d and N549r-d showed very similar patterns of temporal labeling: the majority of the neuronal labeling occurred in the TP, mainly in the TPag and TPg (Figures 8, 9, Table 1). In addition, about 20% of the labeled neurons were found in the ST (area TPO). In N509l-d, more neurons were labeled in the ST (area TPO) and EC than in the TP. In all three cases, the labeled neurons were mostly located in the deep layers of the TP and medial temporal areas (Tables s 2, 3), whereas the labeling of the superficial layers dominated in the ST. Virtually no neurons were labeled in the H, including the subiculum. In N549l-d, the tracer leaked into area 8B, above the cingulate sulcus. In this case, the labeled neurons were more evenly distributed in the TP and medial temporal areas, except for the H. In M2452r-d, the tracer leaked ventromedially and labeled some neurons in the dorsomedial caudate. The majority of the labeled neurons were found in the PRC, and 81% of them were observed in the superficial layers.

### Interconnections Between the pgACC and sgACC

**Figure 10:**
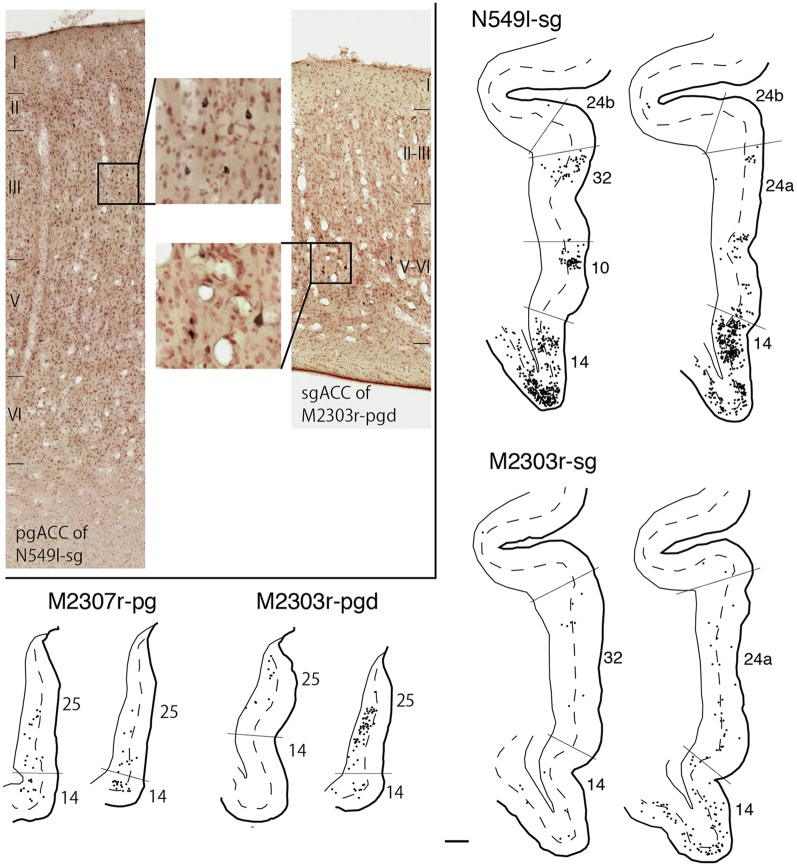
Distribution of the labeled neurons in the pgACC after tracer injection into sgACC (N549l-sg and M2303r-sg), and in the sgACC after injection into pgACC (M2307r-pg and M2303r-pgd). Inset: photomicrographs of the pgACC of N549l-sg (left) and the sgACC of M2303r-pgd (right). Labeled neurons are scattered in the supragranular layers in the former case and in the deep layers in the latter case. Scale bar: 1 mm.

As we and other groups have previously reported, there were direct connections between the pgACC and sgACC (Vogt and Pandya, 1987; Barbas, 1988; Barbas et al., 1999; Joyce and Barbas, 2018; Kim et al., 2018). On the other hand, interconnections between the dACC and sgACC and between the dACC and pgACC were sparse. In the current study, we examined the laminar organization of projections between the pgACC and sgACC. After the tracers were injected into the pgACC, a large majority of the labeled sgACC neurons were observed in the deep layers (45 out of 54 and 48 out of 49 neurons for M2307r-pg and M2303r-pgd, respectively; Figure 10). Following sgACC injections, more labeled neurons were found in the superficial layers of the pgACC (186 out of 233) in one monkey (N549l-sg), and labeled neurons were equally distributed in both the superficial and deep layers in another monkey (superficial: 29 out of 62, M2303r-sg).

## Discussion

To elucidate how sensory information is sent from the temporal cortex to the ACC, we injected neuronal tracers into the three ACC regions and examined the patterns of retrograde labeling in the temporal cortex, including the hippocampus. Previous neurotracing studies have demonstrated that the ACC receives strong inputs from the anterior and medial temporal cortex (Vogt and Pandya, 1987; Barbas, 1988; Carmichael and Price, 1995; Barbas et al., 1999; Saleem et al., 2008; Morecraft et al., 2012; Joyce and Barbas, 2018). To clarify the differences in the input pattern between the three subregions, we compared the distribution of the labeled neurons in the TP and the medial temporal areas between the three injection sites in detail. We especially focused on the differences between the sgACC and pgACC because these areas have been associated with emotion regulation in recent studies (Mayberg et al., 1997; Drevets, 2000; Myers-Schulz and Koenigs, 2012; Wallis et al., 2017; Godlewska et al., 2018).

In addition to the number of the labeled neurons in different areas, the laminar distribution was examined in each area. The laminar origin of the cortico-cortical projection was originally examined in the visual system. Extensive studies have revealed the basic patterns related to the hierarchical organization of the cortical areas: the feedforward (from “lower” areas to “higher” areas) projections originate in the superficial layers, whereas the feedback (from “higher” areas to “lower” areas) projections originate in the deep layers (Rockland and Pandya, 1979; Felleman and Van Essen, 1991). This scheme can be applied to cortical connections, including connections between the frontal and parietal cortices (Felleman and Van Essen, 1991; Webster et al., 1994; Rempel-Clower and Barbas, 2000; Dum and Strick, 2005). In the present study, the laminar origin of the afferent projections from the temporal areas, as well as that of the interconnections within the ACC, indicated that there is a hierarchical organization in the neuronal connections of the ACC.

**Figure 11:**
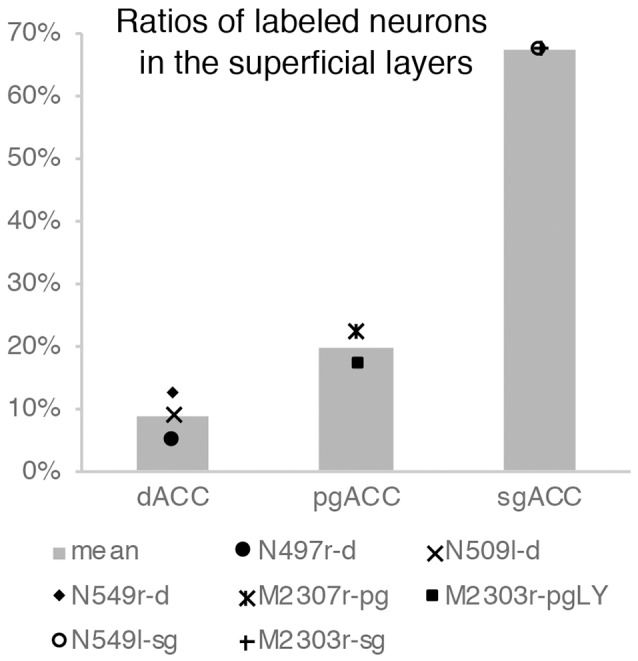
Ratios of labeled neurons in the superficial layers of the TP after tracers were injected into the dACC, pgACC, and sgACC. The graphs show only the results of the cases in which the tracer injection was well confined to the targeted area (see “Results” section).

For injections in all the three ACC regions, many neurons were labeled in the TP areas, especially in the TPag and TPg. Interestingly, after injections into the sgACC, the majority of the labeled TP neurons were located in the superficial layers (Figures 9, 11). In contrast, the majority of the labeled TP neurons were observed in the deep layers after injections into the pgACC or dACC. Our results indicated that TP projections to the sgACC and pgACC/dACC were feedforward and feedback, respectively. The TP is considered to be the highest area in the “ventral stream” of the visual and auditory modarities (Webster et al., 1991; Muñoz-López et al., 2015). For the visual modarity, TP neurons respond to complex visual stimuli, including faces (Nakamura et al., 1994; Nakamura and Kubota, 1996). Neuroimaging studies have demonstrated that the pgACC and sgACC were activated by observation of facial expressions (Blair et al., 1999; Gotlib et al., 2005). Ito et al. (2017) reported that the pgACC activity was correlated with the negativity bias when the subject judged whether the presented faces were happy or sad. Moreover, A recent electrophysiological study from our laboratory revealed that monkey ACC neurons responded to faces and other emotionally significant visual stimuli (Konoike and Nakamura, 2013). The sgACC may extract emotional significance from the highly processed sensory signals in the TP. On the other hand, the TP sends “feedback” type projections to the pgACC and dACC, suggesting that the influence of TP activity on these ACC regions is modulatory.

We also observed labeled neurons in the anterior subiculum, especially in the uncal region, after tracers were injected into the sgACC. In contrast, no/very few neurons were labeled in the hippocampal formation, including the subiculum, after tracers were injected into the pgACC or dACC (Barbas and Blatt, 1995). Previous studies have reported that terminal labeling was observed in the sgACC after injection of an anterograde tracer into the body of the subiculum (Rosene and Van Hoesen, 1977; Aggleton et al., 2015). However, the present retrograde tracing results revealed that the sgACC received more inputs from the uncal region of the subiculum than from the body of the subiculum. A similar pattern of subicular labeling was reported for area 14, an area adjacent to the sgACC (Barbas and Blatt, 1995; Carmichael and Price, 1995). The subiculum is a major output station of the hippocampal formation and sends unidirectional projections to the prefrontal cortex (Rosene and Van Hoesen, 1977; Squire and Zola-Morgan, 1991). After tracers were injected into the sgACC, few neurons were labeled in the medial temporal cortex, including the EC, PRC, and the PHC. In contrast, many neurons were labeled in the EC and PHC after tracers were injected into the pgACC. After injections into the dACC, an appreciable degree of labeling was found in the EC and PRC; however, there were substantial inter-individual differences. In N509l-d, much more neurons were labeled in these areas than in N497r-d and N549r-d. The ST (area TPO) of N509l-d also contained more labeled neurons than in N497r-d and N549r-d. These differences may be because the injection site in N509l-d was more caudal than in the other cases. Labeled cell bodies in the medial temporal areas were mostly observed in the deep layers of the pgACC and dACC, suggesting that the medial temporal-to-ACC projection is of the “feedback” type. The EC, PHC, and PR are known to relay inputs from the sensory and association cortices to the hippocampal formation (Rosene and Van Hoesen, 1977; Squire and Zola-Morgan, 1991). Although previous retrograde and anterograde tracing studies have demonstrated that the EC, PRC, and PHC receive substantial inputs from the pgACC and dACC, these regions receive few inputs from the sgACC (Insausti et al., 1987; Suzuki and Amaral, 1994; Chiba et al., 2001). The present results, together with these previous findings, suggest that the hippocampus receives inputs from the pgACC and dACC (via the medial temporal cortex) and sends outputs to the sgACC. The hippocampus, together with the adjacent medial temporal cortices, is considered to be a center for episodic memory (Squire and Zola-Morgan, 1991). Recent studies have emphasized its role in the memory of relations of multiple objects or objects in a scene rather than its role in the memory of isolated single objects (Alvarado et al., 2002; Lavenex et al., 2006; Browning et al., 2010). This information is important for proper emotional responses in specific situations.

Conversely, previous studies have shown that the pgACC, but not the sgACC, has a reciprocal connection with the dorsolateral prefrontal cortex (DLPFC; Petrides and Pandya, 1999; Morecraft et al., 2012; Joyce and Barbas, 2018; Kim et al., 2018). Thus, the pgACC receives cognitive information from the DLPFC, whereas the sgACC receives sensory and mnemonic information from the temporal lobe. Regarding the interconnections within the ACC, the sgACC sends “feedback” projections to the pgACC, whereas the pgACC sends “feedforward” or “lateral” projections to the sgACC. The pgACC may supply the sgACC with cognitive information from the DLPFC through direct and indirect projections.

After dACC injections, the pattern of retrograde labeling was similar to that following injections into the pgACC; however, unlike the pgACC, many neurons were labeled in the dorsal bank of the superior temporal sulcus (area TPO), where facial expression-related neuronal activities have been previously recorded (Hasselmo et al., 1989). Therefore, this projection may be related to the emotional responses to others’ facial expressions. However, further studies are needed to clarify its specific roles. The input pattern was obviously different between N509l-d and the two other dACC cases (N497r-d and N549r-d). The injection site in N509l-d was more caudal than those in the other cases. Possibly the injection site in N509l-d is the “real” dACC, and those in the other cases may be at the transitional region between the dACC and the pgACC.

**Figure 12:**
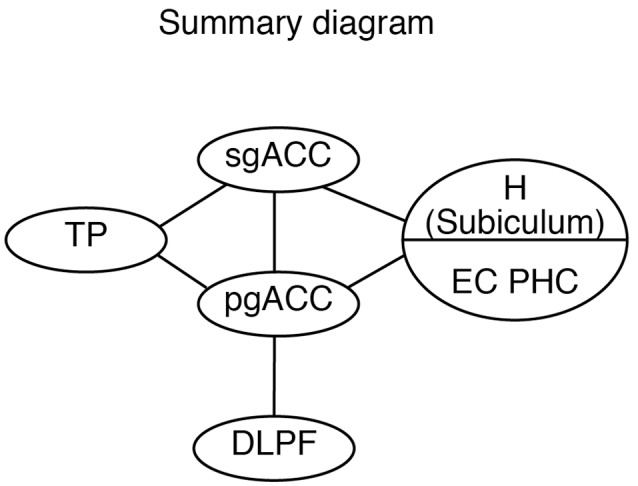
A schematic diagram showing the hierarchical organization of the emotional system, including the pgACC and the sgACC, based on the present and previous studies. DLPFC, dorsolateral prefrontal cortex; EC, entorhinal cortex; H, hippocampus; pgACC, pregenual ACC; sgACC, subgenual ACC; TP, temporal pole.

Although both the sgACC and pgACC are heavily connected within the limbic and autonomic regions of the brain (Kunishio and Haber, 1994; Freedman et al., 2000; Chiba et al., 2001) and are crucial in emotional regulation, the two areas seem to have contrasting roles in emotion regulation (Vidal-Gonzalez et al., 2006; Myers-Schulz and Koenigs, 2012; Wallis et al., 2017; Alexander et al., 2019b). The present study provides the anatomical basis for the differential roles of the sgACC and pgACC: the sgACC is more related to the emotion induction based on the sensory/mnemonic inputs from the TP and hippocampus, whereas the pgACC is more related to cognitive control of emotion depending on the DLPFC (Figure 12).

## Data Availability

The datasets generated for this study are available on request to the corresponding author.

## Ethics Statement

The experiments were conducted according to the Guide for the Care and Use of Laboratory Primates of the Primate Research Institute, Kyoto University (2010). This study was approved by the Animal Welfare and Animal Care Committee of the Primate Research Institute, Kyoto University.

## Author Contributions

HS and YK contributed to data collection and analysis. MN contributed to data analysis and assisted in the preparation of the manuscript. NK contributed to data interpretation and assisted in the preparation of the manuscript. SM contributed to the design of this study, data collection, analysis, and interpretation, and wrote the initial draft of the manuscript. KN contributed to conception and design of this study and also contributed to data interpretation. All authors approved the final version of the manuscript and agree to be accountable for all aspects of the work in ensuring that questions related to the accuracy or integrity of any part of the work are appropriately investigated and resolved.

## Conflict of Interest Statement

The authors declare that the research was conducted in the absence of any commercial or financial relationships that could be construed as a potential conflict of interest.
